# Editorial: Exploring the roles of probiotics/postbiotics and gut microbiota on human health

**DOI:** 10.3389/fphar.2024.1430857

**Published:** 2024-05-23

**Authors:** Cheng Chung Yong, Chyn Boon Wong

**Affiliations:** ^1^ Innovative Research Institute, Morinaga Milk Industry Co., Ltd., Zama, Kanagawa, Japan; ^2^ International Division, Morinaga Milk Industry Co., Ltd., Tokyo, Japan

**Keywords:** probiotics, postbiotics, systemic review, meta-analysis, gut metabolites

The term “probiotics” was first coined in 1965, marking the beginning of a journey that would see these substances, secreted by microorganisms to stimulate the growth of others, become a significant area of scientific research (Lilly and Stillwell, 1965). However, it was in the early 21st century that the study of probiotics truly gained momentum. Researchers, armed with advanced molecular techniques and the power of clinical trials, have since delved into the mechanisms of action, therapeutic potential, and safety of probiotics (Zoumpopoulou et al., 2017). This surge of research has not only fueled interest in probiotics but also opened up new avenues for exploration.

Initially recognized for their positive effects on digestive health, probiotics have emerged as a promising field with potential beyond the gut (Verdu, 2009). Ongoing studies have revealed their potential and ability to address emotional, liver- and metabolic-related health concerns and even improve outcomes for cancer patients (Asseri et al.; Li et al.). Additionally, the modulation of gut microbes has shown potential in alleviating pain by regulating nerve functions (Tian et al.). However, further investigation is needed to fully understand the role of probiotics in pain relief.

Another exciting aspect of probiotics is their interaction with drugs in alleviating disease-relation symptoms, which has been raised up since early 2010 (Stojančević et al., 2014; Panebianco et al., 2018). Exploring the combination effects of probiotics and other compounds can lead to a better understanding of how different components interact to achieve desired effects (Gao et al.; Tian et al.; Wei et al.). By optimizing these combinations, we can harness the full potential of probiotics and other compounds to promote health and wellbeing.

The exponential increase in evidence-based clinical trials with various probiotic strains has made it challenging to select the most appropriate strain for specific diseases. However, the emergence of meta-analysis has facilitated strain evaluation and selection by emphasizing the importance of bacteria specificity (Jin et al.). Additionally, optimizing delivery time, format, dosage, and clinical scale is crucial for obtaining convincing outcomes (Chi et al.).

Since the last decade, incorporating non-viable microorganisms and their components as food supplements or ingredients has gained attention. Numerous terms have been proposed for these non-viable microorganism and/or their components over the past year. In 2021, the International Scientific Association for Probiotics and Prebiotics (ISAPP) unified the term “postbiotics” as “a preparation of inanimate microorganisms and/or their components that confer a health benefit on the host” (Salminen et al., 2021). This perspective article from ISAPP’s members provides valuable insights into the challenges and the need for coherent and harmonized regulatory frameworks for postbiotics (Vinderola et al.).

Overall, the current research on probiotics not only underscores their potential benefits in modulating gut microorganisms and improving human health but also hints at a transformative future. As the field of probiotics continues to evolve, it is essential for us, as a scientific community, to continue exploring their potential beyond the gut. Further research and clinical trials, conducted in collaboration, will help fill the existing knowledge gaps and identify potential challenges in understanding probiotics’ therapeutic potential and their role in improving human health.

In conclusion, probiotics have made remarkable strides since their introduction in 1965. The research conducted in recent years has not only illuminated their expanding potential but also opened up new possibilities for improving health. By harnessing the power of probiotics, we are paving the way for a healthier future, and it is crucial for us all to stay informed and aware of these exciting developments.
